# Divergent genetic mechanisms underlie reversals to radial floral symmetry from diverse zygomorphic flowered ancestors

**DOI:** 10.3389/fpls.2013.00302

**Published:** 2013-08-20

**Authors:** Wenheng Zhang, Victor W. Steinmann, Lachezar Nikolov, Elena M. Kramer, Charles C. Davis

**Affiliations:** ^1^Department of Organismic and Evolutionary Biology, Harvard University HerbariaCambridge, MA, USA; ^2^Department of Biology, Virginia Commonwealth UniversityRichmond, VA, USA; ^3^Centro Regional del Bajío, Instituto de EcologíaPátzcuaro, Mexico

**Keywords:** CYC2-like genes, development, floral symmetry, Malpighiaceae, reversals

## Abstract

Malpighiaceae possess flowers with a unique bilateral symmetry (zygomorphy), which is a hypothesized adaptation associated with specialization on neotropical oil bee pollinators. Gene expression of two representatives of the *CYC2* lineage of floral symmetry TCP genes, *CYC2A* and *CYC2B*, demarcate the adaxial (dorsal) region of the flower in the characteristic zygomorphic flowers of most Malpighiaceae. Several clades within the family, however, have independently lost their specialized oil bee pollinators and reverted to radial flowers (actinomorphy) like their ancestors. Here, we investigate *CYC2* expression associated with four independent reversals to actinomorphy. We demonstrate that these reversals are always associated with alteration of the highly conserved *CYC2* expression pattern observed in most New World (NW) Malpighiaceae. In NW *Lasiocarpus* and Old World (OW) *Microsteria*, the expression of *CYC2*-like genes has expanded to include the ventral region of the corolla. Thus, the pattern of gene expression in these species has become radialized, which is comparable to what has been reported in the radial flowered legume clade *Cadia*. In striking contrast, in NW *Psychopterys* and OW *Sphedamnocarpus*, *CYC2*-like expression is entirely absent or at barely detectable levels. This is more similar to the pattern of *CYC2* expression observed in radial flowered *Arabidopsis*. These results collectively indicate that, regardless of geographic distribution, reversals to similar floral phenotypes in this large tropical angiosperm clade have evolved via different genetic changes from an otherwise highly conserved developmental program.

## Introduction

Convergence is an evolutionary process in which similar features reoccur independently across the Tree of Life (Donoghue, 2005; Protas et al., 2006; Conway Morris, 2008; McGhee, 2011; Wake et al., 2011). These similarities are commonly thought to have arisen as a result of adaptation to similar selective pressures, rather than due to inheritance from a common ancestor. Convergent evolution thus represents an important evolutionary process in shaping the design of organismal diversity. It has been a challenge to explain the origin of convergence, however, since the underlying genetic and developmental bases of this phenomenon remains largely unknown, with notable recent exceptions [reviewed in Gompel and Prud'homme (2009); Conte et al. (2012)]. Evolutionary developmental genetics, however, has facilitated the characterization of several types of convergence operating at the genetic level. In some cases, studies have revealed convergence in terms of genetic change (e.g., Sucena et al., 2003; Prud'homme et al., 2006; Rosenblum et al., 2010), but in others, distinct genetic mechanisms were uncovered (e.g., Hoekstra and Nachman, 2003; Wittkopp et al., 2003; Steiner et al., 2009). A special case of convergence is reversion, the reappearance of ancestral phenotypes. Here, we use the flowering plant family Malpighiaceae, which has been the subject of recent floral developmental genetic studies (Zhang et al., 2010, 2012), to investigate the genetic patterns associated with four independent examples of reversion to an ancestral floral phenotype. We also discuss the diverse evolutionary trajectories underlying these independent reversions.

**Figure 1:**
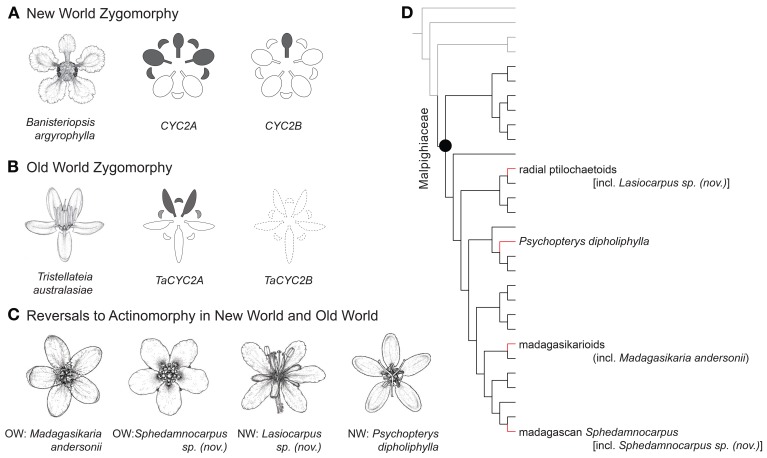
**Phylogenetic positions, floral morphology, and *CYC2* expression of Malpighiaceae. (A)** *Banisteriopsis argyrophylla* illustrates the stereotypical NW floral morphology and pattern of *CYC2* expression in NW Malpighiaceae (expression shown in gray). **(B)** *Tristellateia australasiae* represents the OW flowers that have departed from NW morphology with two dorsal petals (*CYC2B* is missing shown as dash line). **(C)** *Madagasikaria andersonii*, *Lasiocarpus sp. (nov.)*, *Psychopterys dipholiphylla* and *Sphedamnocarpus sp. (nov.)* represent four OW floral phenotypes that have evolved independently from a similar NW type ancestor. **(D)** Phylogeny indicating relationships of the four focal clades: radial ptilochaetoids [including *Lasiocarpus sp. (nov.)*], *Psychopterys dipholiphylla*, madagasikarioids (including *Madagasikaria andersonii*) and Malagasy *Sphedamnocarpus* [including *Sphedamnocarpus sp. (nov.)*]. We examined the representatives of these four clades in this study. Gray lines highlight the radially symmetrical sister groups of Malpighiaceae, i.e., Centroplacaceae and Elatinaceae; black lines highlight Malpighiaceae species with the stereotypical NW floral morphology; red highlights the three OW clades with floral morphologies reversed to radially symmetry. The phylogenetic relationships inferred here are highly supported by previous studies (Davis et al., 2001; Davis and Chase, 2004; Wurdack and Davis, 2009; Davis and Anderson, 2010; Xi et al., 2012). Note: floral line drawings are not proportional to the sizes of flowers.

Malpighiaceae are a family of tropical trees, shrubs, and vines of both the New World (NW) and Old World (OW) tropics and subtropics. The zygomorphic (bilaterally symmetrical) floral morphology of the more than 1000 NW species of this clade is very distinctive and highly conserved, especially with regard to symmetry and pollinator reward (Figure 1A). The NW Malpighiaceae flower has a single upright dorsal banner petal that is strongly differentiated from other petals in the corolla whorl, and appears to orient and attract an extremely limited suite of pollinators, principally female bees of the tribes Centridini, Tetrapedini, and Tapinotaspidini (Vogel, 1974). These bees use their mandibles to grasp the base of this distinct dorsal banner petal and then use their fore and mid legs to access the oil glands borne in pairs on the abaxial surface of the sepals. It is thought that the stereotypical floral morphology of NW Malpighiaceae is maintained primarily due to this specialization on oil-bee pollinators (Vogel, 1974; Anderson, 1979).

We recently established the likely genetic basis for the origin of this floral zygomorphy in NW Malpighiaceae, especially in regards to the unique banner petal morphology (Zhang et al., 2010, 2012). *CYCLOIDEA2*-like (*CYC2*) transcription factors of the ECE clade within the TCP gene family have been shown to be critical for establishing floral symmetry and to have been repeatedly recruited to regulate floral zygomorphy in diverse angiosperm lineages [reviewed in Howarth and Donoghue (2006); Preston and Hileman (2009); Citerne et al. (2010)]. The common finding in all of these studies, using model species with functional data and correlative patterns of gene expression from non-model species, is the persistent expression of *CYC2* homologs in dorsal floral organs [reviewed in Preston and Hileman (2009); Citerne et al. (2010)]. Moreover, *CYC2* loss-of-function mutants exhibit a fully actinomorphic (radially symmetrical) phenotype in which all floral organs gain ventral floral organ identity (Luo et al., 1996; Cubas et al., 1999b; Busch and Zachgo, 2007), or exhibit greatly reduced dorsal floral organ identity (Feng et al., 2006; Broholm et al., 2008). Conversely, ectopic expression of *CYC*, such as in the *backpetals* mutant of *Antirrhinum*, produces a radialized phenotype due to dorsalization of the corolla (Luo et al., 1999).

In Malpighiaceae, two clades of *CYC2* were identified, *CYC2A* and *CYC2B*, which resulted from a duplication event coincident with the origin of the family (Zhang et al., 2010). In most NW species these loci are differentially expressed along the dorsoventral axis such that *CYC2A* is expressed in the dorsal banner petal and two adjacent lateral petals while *CYC2B* is restricted solely to the banner petal (Figure 1A). This pattern of *CYC2* expression is conserved across three distantly related NW species [*Janusia guaranitica* A. Juss., *Byrsonima crassifolia* Kunth (Zhang et al., 2010), and *Bunchosia glandulifera* (Jacq.) Kunth (Zhang et al., 2012)], that span the origin of the family and its unique floral morphology (Davis and Anderson, 2010; Zhang et al., 2010, 2012). These results indicate that *CYC2* genes likely play a key role in this plant pollinator mutualism and are thus good candidates for understanding the genetic basis of derived floral symmetries in Malpighiaceae.

**Figure A1:**
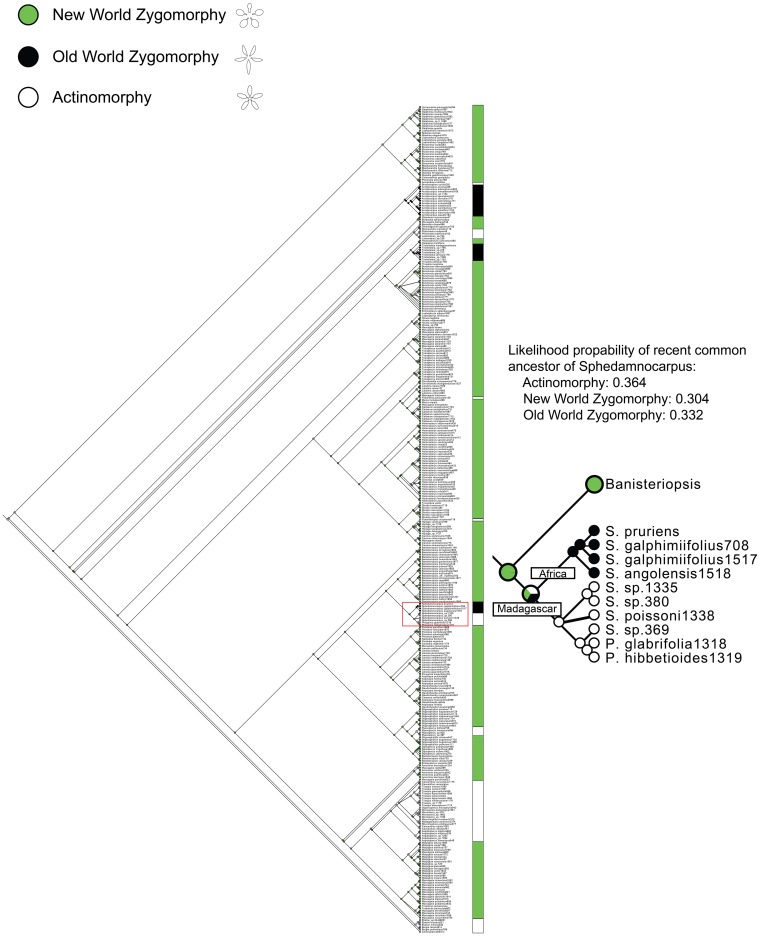
**Ancestral character state reconstruction of floral symmetry.** ML analysis indicates the NW zygomorphy has been lost nine times in Malpighiaceae. OW zygomorphy has evolved from an NW ancestor three times in parallel; the NW zygomorphy has also been reversed to actinomorphy eight times in the family. The part of tree is enlarged to show the evolutionary transition of floral symmetry in Sphedamnocarpus. These results indicate that the ancestral character state reconstruction suggests the common ancestor of the Sphedamnocarpus is equivocal among NW zygomorphy, OW zygomorphy, or actinomorphy. The comparative expression data help to distinguish between these scenarios (see Discussion).

The oil bees that pollinate NW Malpighiaceae are absent in the OW (Vogel, 1974; Michener, 2000), which is particularly interesting because most OW Malpighiaceae lack the oil glands and floral banner petals (Vogel, 1990; Davis, 2002; Davis et al., in review) that appear to be critical to the NW pollination syndrome (Anderson, 1979). These OW species possess either an altered form of floral zygomorphy with two dorsal petals or completely actinomorphic flowers (Figure 1) (Davis and Anderson, 2010). Recent phylogenetic studies suggest that these derived forms of zygomorphy and actinomorphy evolved three and eight times, respectively (Figure A1) (Davis and Anderson, 2010; Zhang et al., 2010, 2012). Two of the eight reversals to actinomorphy may have evolved from an ancestor with the altered form of floral zygomorphy (Figure A1) (Davis and Anderson, 2010; Zhang et al., 2010, 2012). The only obvious pollinator rewards of these atypical Malpighiaceae are pollen, which likely reflects their shifts to new pollinators (Lobreau-Callen, 1989; Davis, 2002). Malpighiaceae therefore represent a set of natural experiments involving replicate clades that initially possessed the ancestral zygomorphic NW floral morphology but diverged dramatically in the absence of their specialist oil-bee pollinators.

Recently, we built upon the genetic and developmental framework established for NW Malpighiaceae to examine parallel evolution of three OW Malpighiaceae lineages that evolved the altered form of zygomorphy described above (Figure 1B) (Zhang et al., 2012). In all three cases, the species exhibit a loss of *CYC2B* function, and a strikingly similar shift in the expression of *CYC2A* that is coincident with their shift in floral symmetry. These results indicate that similar floral phenotypes of OW Malpighiaceae have evolved via parallel genetic changes from an otherwise highly conserved developmental program. Interestingly, in two of these three zygomorphic OW clades, *Sphedamnocarpus* and *Acridocarpus-Brachylophon*, this altered form of zygomorphy is closely related to actinomorphic species (Figure A1) (Davis and Anderson, 2010; Zhang et al., 2010), which facilitates the extension of our studies into additional forms of symmetry.

Here, we continue with a similar line of inquiry, but turn our attention to investigate genetic modifications to this conserved developmental program that are associated with reversals to floral actinomorphy in four clades of Malpighiaceae. In addition to our analysis of two OW genera, two NW clades that have also independently lost the typical NW floral morphology and have become actinomorphic were also analyzed. These represent four phylogenetically independent contrasts to investigate reversals to actinomorphy from zygomorphic flowered ancestors that are also geographically distinct. We show that the conserved *CYC2* program in the NW zygomorphic flowers is modified in each of the four actinomorphic flowered clades: *Microsteira* and *Sphedamnocarpus* from the OW, and *Psychopterys* and *Lasiocarpus* from the NW (Figures 1C,D). Our results indicate that these reversals in floral symmetry are the result of distinct modifications to the conserved NW *CYC2* program, including the loss of *CYC2* expression in Malagasy *Sphedamnocarpus* and NW *Psychopterys*, and the radialized expansion of expression in OW *Microsteira* and NW *Lasiocarpus*. Thus, *CYC2*-like genes seem to play diverse roles in the evolution of floral symmetry in Malpighiaceae. The loss of banner petal-associated *CYC2B* expression is particularly common, but has been combined in different ways with either shifts to radialized expression or complete loss of *CYC2A* expression, consistent with each type of symmetry. By analyzing these patterns of *CYC* expression in an evolutionary context, we gain a comprehensive understanding of the diversification of this genetic program. Within this single tropical flowering plant clade we find that essentially all potential mechanisms for facilitating shifts to actinomorphy are observed, highlighting the context-dependent and stochastic nature of developmental evolution.

## Results

### *CYC2*-like gene evolution in the four derived actinomorphic clades

**Figure 2:**
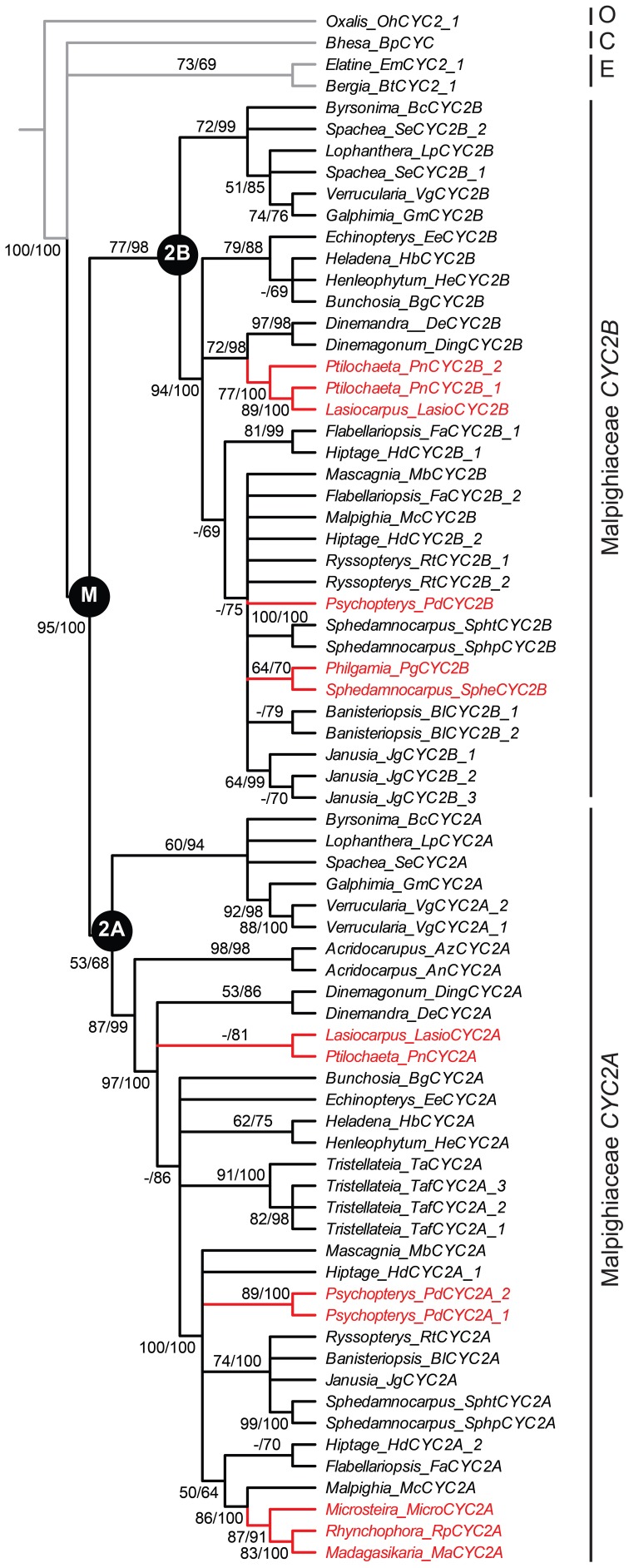
**Phylogeny of *CYC2*-like genes for Malpighiaceae with the four reversal lineages highlighted.** Bayesian majority rule consensus topology shown; clades with ≥50% maximum likelihood (ML) bootstrap support and ≥60% Bayesian posterior probabilities depicted above lines, respectively. ML bootstrap support <50% indicated with a hyphen. Inferred gene tree is reflective of accepted species tree relationships (Davis and Anderson, 2010). Accessions highlighted in red include the four Malpighiaceae clades examined here that exhibit reversal floral phenotypes—madagasikarioids and Malagasy *Sphedamnocarpus* in OW, and radial ptilochaetoids and *Psychopterys dipholiphylla* in NW. See Materials and Methods and Table A1 for species identities and voucher information. C, Centroplacaceae; E, Elatinaceae; M, Malpighiaceae; O, Oxalidaceae.

**Figure A2:**
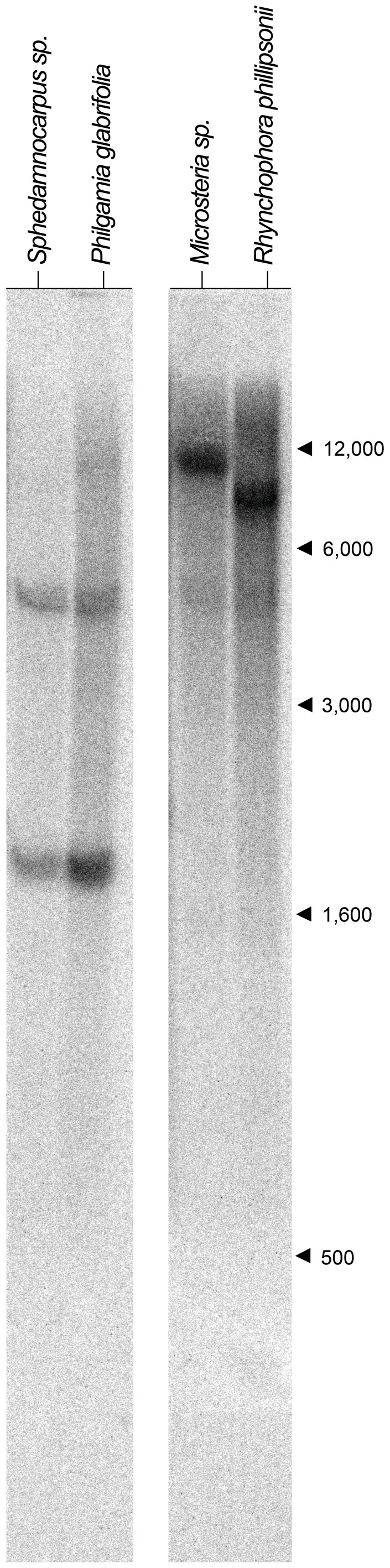
***CYC2*-like gene Southern hybridization results for *Sphedamnocarpus sp*.,*Philgamia glabrifolia*,*Microsteria sp*., and *Rhynchophora phillipsonii*.** Restriction digests using *EcoRI* are shown for *Sphedamnocarpus sp*.,*Philgamia glabrifolia*,*Microsteria sp*., and *Rhynchophora phillipsonii*. Arrows and numbers indicate molecular size markers (in base pairs).

We first identified *CYC2*-like homologs from eight species using degenerate primers and exhaustive screening of PCR clones as described in the Materials and Methods and in Zhang et al. (2010); Zhang et al. (2012) (Table A1). These species represent the four Malpighiaceae lineages that have reverted to actinomorphy: the radial flowered OW madagasikarioid clade (represented by *Madagasikaria andersonii* C. Davis, *Microsteira sp*., and *Rhynchophora phillipsonii* W. R. Anderson), the OW Malagasy *Sphedamnocarpus* clade [represented by *Philgamia glabrifolia* Arènes, and *Sphedamnocarpus sp. (nov.)*], the NW *Psychopterys* clade (represented by the only species *Psychopterys dipholiphylla* (Small) W. R. Anderson and S. Corso), and the NW ptilochaetoid clades (represented by *Lasiocarpus sp*. and *Ptilochaeta nudipes* Griseb.). The phylogenetic positions of these *CYC2* homologs were inferred using our previously published *CYC2* homologs, which sampled broadly across Malpighiaceae, including taxa that are closely related to the additions here (Zhang et al., 2010, 2012). Phylogenetic relationships inferred from *CYC2* mirror our understanding of accepted species tree relationships (Davis et al., 2001, 2002; Davis and Anderson, 2010). *Lasiocarpus, Ptilochaeta*, and *Psychopterys dipholiphylla* maintain both copies of *CYC2A* and *CYC2B*, but in contrast, *CYC2B* was not detected in the madagasikarioid clade where we sampled *Madagasikaria andersonii* C. Davis, *Microsteria sp*., and *Rhynchophora phillipsonii* W. R. Anderson. (Figure 2, Table A1) (Zhang et al., 2010). These results were confirmed by Southern analyses in *Microsteria sp*. and *Rhynchophora phillipsonii* (Figure A2). Meanwhile, the *CYC2A* in two radialized species of Malagasy *Sphedamnocarpus* [i.e., *Philgamia glabrifolia* Arènes and *Sphedamnocarpus sp. (nov.)*], could not be amplified by PCR, but Southern analyses identified more than one copy of *CYC2* (Figure A2). It is worth noting that in our previous paper (Zhang et al., 2012), we examined an African *Sphedamnocarpus*, a close relative of Malagasy *Sphedamnocarpus*, which exhibits a greatly altered zygomorphy from most NW species. *CYC2A* and *CYC2B* were both identified with no bias from this African species. These results suggest that *CYC2A* in species of Malagasy *Sphedamnocarpus* has diverged such that we cannot easily isolate it via degenerate PCR from genomic DNA or cDNA.

### Loss of *CYC2* expression in two derived actinomorphic lineages

**Figure 3:**
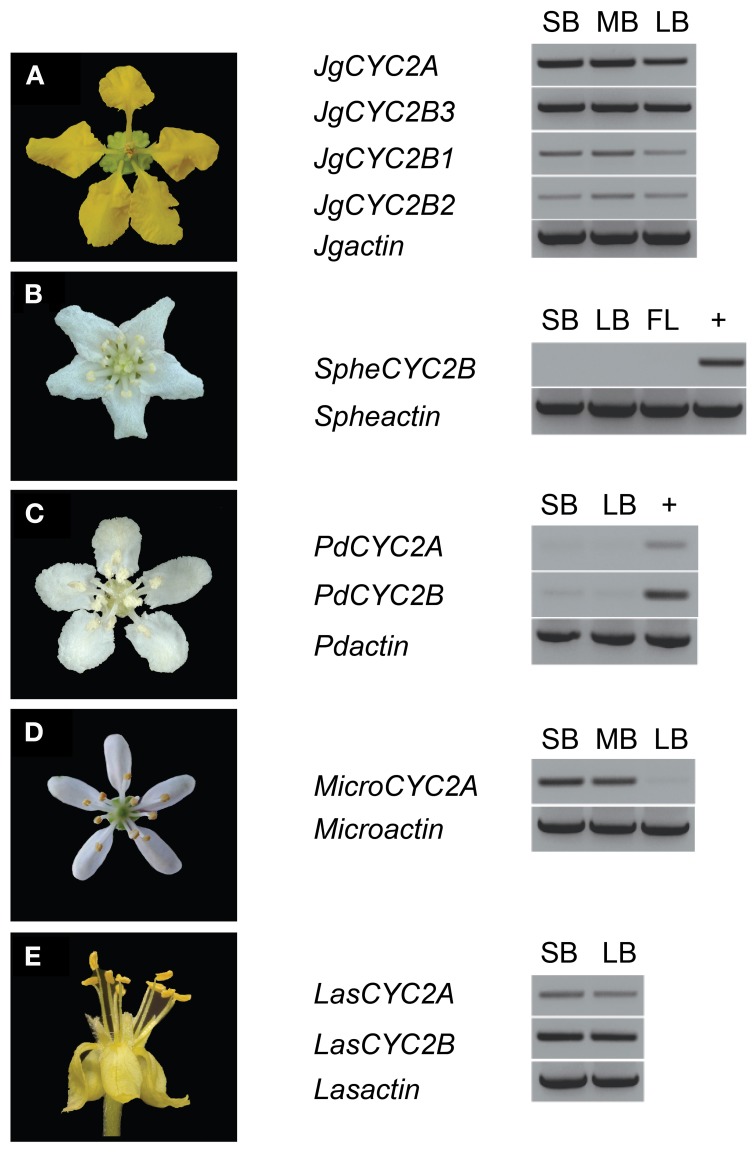
**Locus-specific RT-PCR for *CYC2*-like gene expression in Malpighiaceae. (A)** *Janusia guaranitica* shows the consistent *CYC2A* and *CYC2B* expression in NW Malpighiaceae. **(B–E)** The temporal pattern of *CYC2* expression in the derived actinomorphic Malpighiaceae *Sphedamnocarpus sp*. **(B)** *Psychopterys dipholiphylla* **(C)** *Microsteria sp*. **(D)** *Lasiocarpus sp*. **(E)** +, genomic DNA was used as a control for PCR efficiency. *MicroCYC2B* and *SpheCYC2A* were not included in the analyses due to failure to recover these loci using exhaustive PCR and clone screening described in the Materials and Methods. *ACTIN*-specific primers were used as a positive control (Zhang et al., 2010). Abbreviations are as follows: SB, small buds ~10–20% of full size buds; MB, medium buds ~40–60% of full size buds; LB, large buds ~70–90% of full size buds; FL, open flowers.

**Figure 4:**
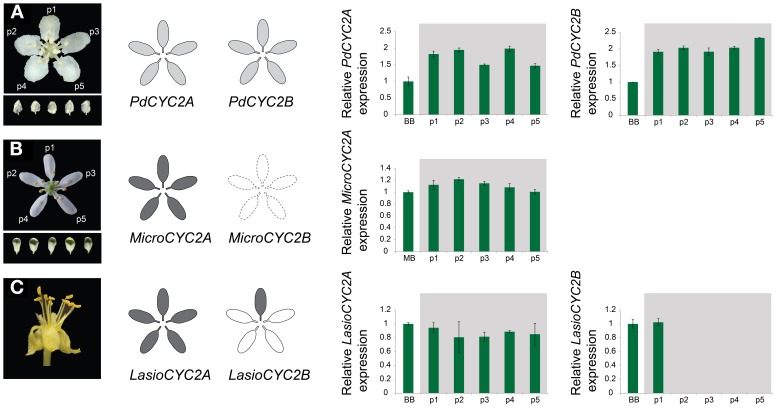
**Quantitative RT-PCR (qRT-PCR) expression of *CYC2*-like genes for the reversed floral morphologies in the Malpighiaceae *Psychopterys dipholiphylla* (A), *Microsteria sp*. (B), and *Lasiocarpus sp*. (C).** *MicroCYC2B* was not included in this analysis due to inability to recover it from *Microsteria* sp. Grayscale shading on floral diagrams summarizes the relative strength of the spatial pattern of *CYC2* expression in the corolla. qRT-PCR expression data was determined for dissected floral organs at mid to late stage (also see Materials and Methods). Expression levels are relative to the control β*-tubulin*. Error bars represent standard errors. p1–p5, five dissected petals from a single flower. Levels of gene expression among p1-p5 are not significantly different (*P* > 0.05) for *PdCYC2A*, *PdCYC2B*, *MicroCYC2A*, and *LasioCYC2A*.

To examine whether these *CYC2* homologs are involved in floral development, locus-specific reverse transcription (RT)-PCR was carried out at several stages of floral development (Figure 3). Most NW Malpighiaceae express both *CYC2A* and *CYC2B* consistently throughout floral development (Figure 3A) (Zhang et al., 2010, 2012). The late stage expression of *CYC2* like genes has been shown to be critical for developing zygomorphic flowers in many lineages (Luo et al., 1996, 1999; Cubas et al., 1999a; Feng et al., 2006; Busch and Zachgo, 2007; Broholm et al., 2008; Wang et al., 2008). In sharp contrast, RT-PCR revealed that *CYC2* homologs are not expressed in the OW *Sphedamnocarpus sp. (nov.)* floral buds and show exceptionally low activity in the NW *Psychopterys dipholiphylla*, especially at late stages of floral development (Figures 3B,C). We used quantitative RT-PCR to further examine the spatial pattern of *CYC2* expression in actinomorphic flowered *Psychopterys dipholiphylla*. This experiment revealed that the low expression levels of *CYC2A* and *CYC2B* that are present have become more radialized by expanding into the ventral region of the corolla (Figure 4A). However, both of these actinomorphic flowered lineages show greatly down regulated *CYC2* expression: *CYC2* expression is more radialized in the corolla of *Psychopterys dipholiphylla* (Figure 4A), but at barely detectable levels (Figure 3C); while, *Sphedamnocarpus sp*. expresses no *CYC2* during floral development (Figure 3B).

### Radialization of *CYC2* expression in two derived actinomorphic lineages

In the OW *Microsteria sp*. and the NW *Lasiocarpus sp*., *CYC2* is expressed at a high level throughout floral development (Figures 3D,E). Moreover, quantitative RT-PCR revealed that *CYC2A* expression is expanded to include the ventral region for *Microsteria sp*. and *Lasiocarpus sp*. (Figures 4B,C). While *MicroCYC2A* and *LasCYC2A* expression is similarly high across the entire corolla, *LasCYC2B* is expressed only in the innermost single petal in *Lasiocarpus sp*. Species of Malpighiaceae have very conserved floral aestivation, in which the innermost petal in the corolla always forms the conspicuous dorsal banner petal in the NW species (Eichler, 1878; Zhang et al., 2010). This suggests that *LasCYC2B* is expressed in the petal that is homologous to the banner petal of most NW Malpighiaceae. The OW madagasikarioids and the NW radial ptilochaetoids therefore share very similar changes in the pattern of *CYC2A* expression by expanding gene expression such that it is equally distributed in all five petals.

## Discussion

### Divergent genetic patterns underlie reversion to actinomorphy in malpighiaceae

**Figure 5:**
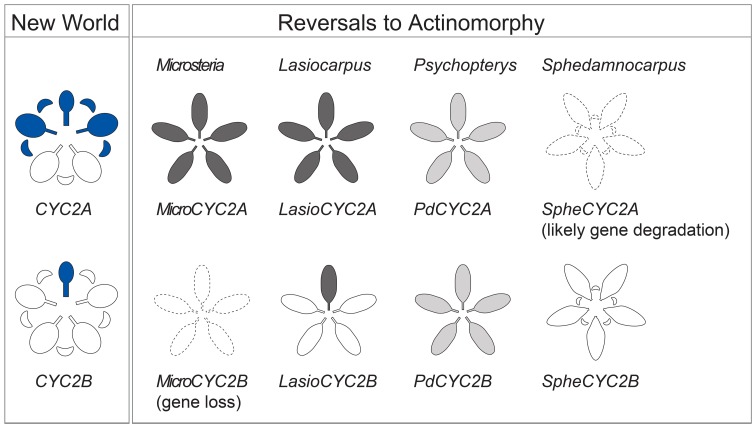
**Summary of *CYC2*-like gene expression.** Expression of *CYC2*-like genes in NW Malpighiaceae and in reversals to actinomorphy in the four Malpighiaceae clades represented, from left to right, by *Microsteria sp*., *Lasiocarpus sp*., *Psychopterys dipholiphylla*, and *Sphedamnocarpus sp*. The blue shading of the NW Species indicates late stage *CYC2* gene expression (Zhang et al., 2010, 2012). The gradient shading in the derived actinomorphic species, from white to dark gray, indicates increasing intensity of *CYC2* expression, respectively. The dotted outlines indicate the loss of gene copy, or inability to recover this gene due to its likely degradation.

We observed that the conserved *CYC2* pathway of the stereotypical NW Malpighiaceae changes dramatically in four clades that have each experienced reversals to floral actinomorphy. Interestingly, this occurs against two strikingly different biogeographical backgrounds. Two actinomorphic clades on opposite sides of the Atlantic Ocean, the OW *Microsteira sp*. and NW *Lasiocarpus sp*., shift their expression to be broad, whereas the similarly distributed OW Malagasy *Sphedamnocarpus* and NW *Psychopterys* lack late stage *CYC2*-like gene expression (Figure 5). These contrasting patterns seem to reflect the two general *CYC2*-dependent mechanisms that may be responsible for the transition to actinomorphy from a zygomorphic ancestor: (i) ectopic *CYC2* expression resulting in phenotypic dorsalization of the entire flower, or (ii) loss of function resulting in phenotypic ventralization. However, we cannot exclude the possibility that *CYC2* downstream factors may also play a role for floral radialization. The presence of both patterns underscores the fact that each of these four clades independently transitioned to actinomorphy in response to the loss of their specialist NW oil bee pollinators, traversing distinct genetic trajectories in the process.

The evolutionary potential for such modification of a pre-existing *CYC*-dependent program was immediately recognized in early studies of *CYC* mutants in *Antirrhinum* (Coen and Nugent, 1994; Coen et al., 1995). However, while loss-of-function represents an attractively simple model for reversals to actinomorphy, Donoghue et al. (1998) pointed out that a gain of function model in which expression is expanded into the ventral petals seems more likely from a morphological perspective. Diverse studies have, in fact, revealed both patterns. Ventral expansion of *CYC2* expression has been observed in the actinomorphic flowers of the legume *Cadia* (Citerne et al., 2006) and in *Tengia*, a member of the Gesneriaceae (Pang et al., 2010). Conversely, a different actinomorphic Gesneriaceae, *Bournea leiophylla*, exhibits rapid down-regulation of *CYC2* expression at later developmental stages (Zhou et al., 2008), suggesting loss-of-function. Similarly, the actinomorphic flowers of *Plantago*, a member of the Plantaginaceae, are associated with degeneration of both the *CYC*-based dorsal identity and MYB-based ventral identity programs, although one *CYC2* homolog is still broadly expressed (Preston et al., 2011).

### Synthesis of floral symmetry evolution in malpighiaceae

**Figure 6:**
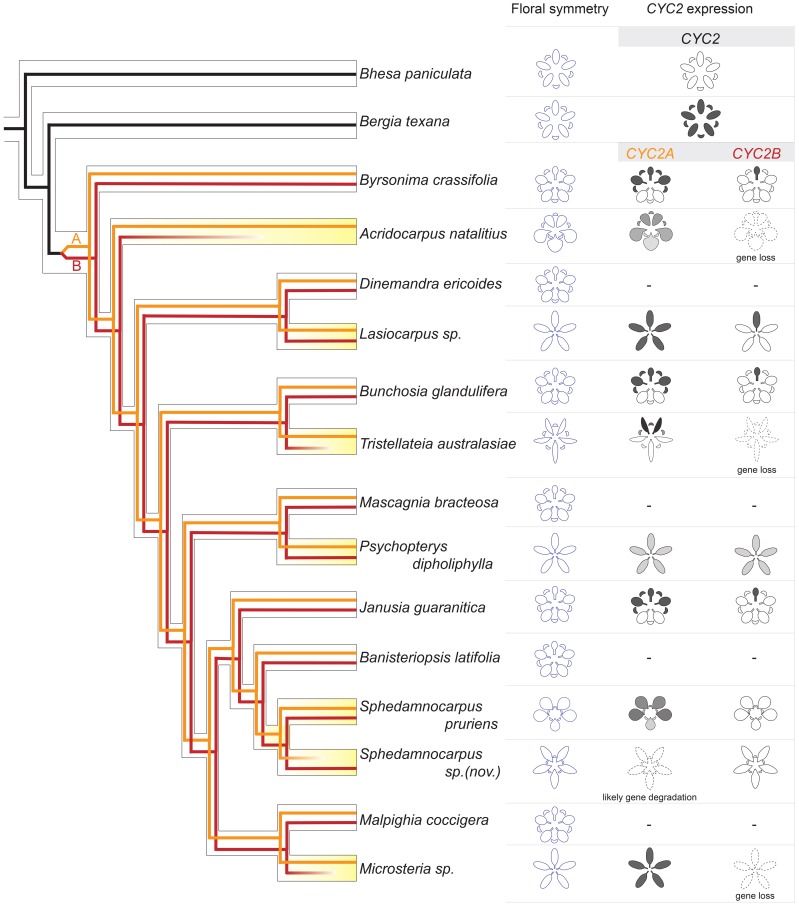
**A synopsis of *CYC2* evolution underlying floral symmetry evolution in Malpighiaceae and close relatives.** The hollow-branch phylogeny indicates species tree relationships based on Davis and Anderson (2010). The solid-line phylogeny indicates *CYC2* gene tree, which was embedded within the species tree. Black lines: the *CYC2* gene lineage in close relatives, Centroplacaceae (e.g., *Bhesa paniculata*) and Elatinaceae (e.g., *Bergia texana*); dark red and orange lines: *CYC2A* and *CYC2B*, respectively. *CYC2* paralogs originated through a gene duplication that occurred in the common ancestor of all Malpighiaceae. Most species of NW Malpighiaceae have maintained both *CYC2A* and *CYC2B* copies, while many of their OW counterparts (e.g., *Acridocarpus natalitius, Tristellateia australasiae, Sphedamnocarpus sp. (nov.)*, and *Microsteria sp*.) lost one of the two *CYC2* copies indicating as fainting out lines. The expression patterns of *CYC2* genes at late stages of floral development is summarized for all of the sampled outgroup and Malpighiaceae taxa: the radial outgroup genera *Bhesa paniculata* and *Bergia texana*; three zygomorphic species of NW Malpighiaceae that are phylogenetically distant related and span the origin of Malpighiaceae, *Bunchosia glandulifera, Byrsonima crassifolia*, and *Janusia guaranitica*; and several species that lost the NW zygomorphy, which are highlighted in yellow. OW species: *Acridocarpus natalitius, Sphedamnocarpus pruriens, Sphedamnocarpus sp.(nov.)*, and *Tristellateia australasiae*; NW species: *Lasiocarpus sp.*, and *Psychopterys dipholiphylla*. A dash indicates NW counterparts for which *CYC2* genes were cloned but the expression data is not available.

Currently, we have data on *CYC2* homolog expression patterns from ten major clades within Malpighiaceae: three with the stereotypical NW zygomorphy, three with the altered form of OW zygomorphy, and four that are completely actinomorphic. Analyzing these results in a phylogenetic context provides insight into the major evolutionary events associated with diversification of floral morphology across the family (Figure 6). The origin of zygomorphy in the NW ancestors of the family appears to be closely associated with a duplication event that gave rise to the *CYC2A* and *2B* lineages. Analyses of divergent taxa with the distinct NW zygomorphy reveal a pattern in which *CYC2A* is expressed in the banner petal and two lateral petals while *CYC2B* is narrowly confined to the banner petal alone (Figure 6) (Zhang et al., 2010, 2012). Similar to what has been observed in many other systems [reviewed in Citerne et al. (2010)], the expression of these *CYC2* paralogs in partially overlapping domains may create a dosage-based mechanism whereby the banner petal is distinguished by the highest concentration of *CYC2A*+*B*, followed by the lateral petals with *CYC2A* alone, and the ventral petals expressing no *CYC2* homologs. The origin of this program in the Malpighiaceae, however, is clouded by the presence of two contrasting patterns in their closest actinomorphic flowered relatives (Figure 6) (Zhang et al., 2010). Reconstruction of the ancestral condition is equivocal as to whether absence of expression or broad expression is ancestral (Zhang et al., 2010). Although the close radial flowered relative of Malpighiaceae, *Bergia* (Elatinaceae), may seem atypical in having broad *CYC2* expression, a strikingly similar pattern has been observed in the asterid clade Dipsacales (Howarth et al., 2011). In this case, broad *CYC2* expression in actinomorphic *Viburnum* appears to be the ancestral condition predating evolution of dorsal-restricted expression in the zygomorphic Caprifoliaceae. However, these two examples in the Malpighiales and Dipsacales highlight the general lack of both expression and functional data from actinomorphic flowers, and raise the possibility that the *Arabidopsis* pattern, which has been assumed to represent the ancestral actinomorphic condition (Cubas et al., 2001), may not actually be the best representative. More research is needed on *CYCLOIDEA* function and expression in radial flowered angiosperm clades.

If we turn our attention back to the clades in which the NW zygomorphy is lost, we see that these transitions are consistently associated with extreme reduction or loss of *CYC2B* expression. The only exception in this regard is *Lasiocarpus*, where it seems likely that the radial, strong expression of *CYC2A* negates the remaining banner petal-specific expression of *CYC2B*. Interestingly, this pattern directly parallels what has been observed in the actinomorphic legume *Cadia*, where expression of one *CYC2* copy has been maintained at weak to moderate expression levels in dorsal petals, while the second copy is broadly expressed at very high levels (Citerne et al., 2006). Considering *CYC2A* expression patterns, there is a much greater diversity in patterns of expression across clades, but a highly consistent correlation with floral morphology. When the NW-type zygomorphy is altered via a shift to two dorsal petals instead of one, the axis of *CYC2A* expression shifts in concert, although this new expression domain can be arrayed in a differential gradient across the corolla (i.e., *Acridocarpus* and *Sphedamnocarpus puriens*) or tightly restricted to the two dorsal petals (i.e., *Tristellateia*) (Figure 6; Zhang et al., 2012). When actinomorphy evolves, the pattern of expression is much more variable, but still completely consistent with morphology. Although we might be tempted to conclude that *Lasiocarpus* and *Microsteria* flowers are essentially dorsalized while those of *Psychopterys* and *Sphedamnocarpus sp.(nov.)* are ventralized, this is difficult to determine without information on other aspects of radial organ identity. In the *Plantago* case described above, data on the *CYC2* loci alone might lead to a conclusion that the flowers are dorsalized, but the complete absence of downstream components of both the dorsal and ventral identity pathways suggests that they are actually lateralized (Preston et al., 2011). Unfortunately, there is no current data to suggest that the functions of these downstream factors are conserved outside the Lamiales, so we have no candidates for effectors of *CYC2* function in Malpighiales. What is clear is that despite three distinct floral forms, the expression of *CYC2* homologs correlates incredibly well with floral morphology across Malpighiaceae, providing further evidence that these genes play crucial roles in the development and evolution of floral symmetry in this clade.

### Evolutionary transition of floral symmetry in *sphedamnocarpus*

In addition to illuminating broader mechanisms of morphological evolution, our molecular studies have helped us better understand the small scale character state transitions that gave rise to divergent floral forms across Malpighiaceae, particularly in *Sphedamnocarpus*. This OW genus is comprised of two major clades: an African clade represented by *S. puriens*, which bears yellow or cream-colored flowers with the altered NW zygomorphy, and a Malagasy clade represented by *S. sp. (nov.)*, which bears white actinomorphic flowers (Davis and Anderson, 2010). The closest sister genus of *Sphedamnocarpus* in the NW is *Banisteriopsis* C. B. Rob, whose yellow flowers have the typical banner petal zygomorphy (Davis and Anderson, 2010). Simple ancestral character state reconstruction of floral symmetry cannot resolve whether the *Sphedamnocarpus* ancestral state was NW zygomorphy, the altered OW zygomorphy, or actinomorphy (Figure A1), but a much clearer evolutionary scenario becomes apparent with the addition of our molecular data. Both *Sphedamnocarpus* species lack *CYC2B* expression, indicating that this was most likely the case in their common ancestor. Given that loss of *CYC2B* expression is closely associated with loss of the NW zygomorphic pattern, we would hypothesize that the common ancestor of all *Sphedamnocarpus* did not possess the banner petal morphology. Furthermore, since the actinomorphy of *S. sp. (nov.)* is associated with complete loss of *CYC2A*, it seems unlikely that this state represents the ancestral condition (i.e., a scenario that would invoke the re-evolution of the zygomorphic pattern of *CYC2* gene expression in zygomorphic African *Sphedamnocarpus*). Thus, we can propose a model for the stepwise reversion to actinomorphy in this lineage. First, *CYC2B* expression was lost in the OW ancestor, most likely in concert with the evolution of the altered form of zygomorphy. This ancestor then gave rise to the African clade with its shifted axis of graded differential *CYC2A* expression, as well as to the Malagasy clade in which loss of *CYC2A* facilitated the transition to complete actinomorphy. This is the most parsimonious explanation of the current data, but further sampling of this fascinating lineage will allow our hypothesis to be further tested and refined as needed. Thus, the *Sphedamnocarpus* clade represents a very nice example of the way in which phylogenetically focused studies of floral gene expression can illuminate otherwise uncertain ancestral character state reconstructions involving important morphological innovations.

## Conclusions

Taken together, our study illustrates both the constrained and contingent nature of evolution. While certain patterns are fairly predictable, such as loss of *CYC2B* function in association with loss of the stereotypical NW zygomorphy, nothing is absolutely certain, as evidenced by the retention of *CYC2B* in *Lasiocarpus*. There are alternative genetic solutions to the problem of shifting pollinator availability, even when the morphological outcomes appear convergent. In this way, the Malpighiaceae offer a fascinating context in which to study the intersection between biogeography, pollinator interactions, floral morphology, and genetic evolution. Further studies will continue to build on and expand these models, hopefully providing a clearer picture of the complex evolutionary processes at work during the diversification of the family.

## Materials and methods

### Specimen collections

Specimens of *Microsteria sp*. and *Rhynchophora phillipsonii* W. R. Anderson are from Toliara, Madagascar; *Philgamia glabrifolia* Arènes from Mountain Ibity, Fianarantsoa, Madagascar; *Sphedamnocarpus sp.(nov.)* from Isalo National Park, Fianarantsoa, Madagascar; *Lasiocarpus sp*. from the municipio de Huitzuco de los Figueroa, Guerrero, Mexico; and *Psychopterys dipholiphylla* (Small) W. R. Anderson and S. Corso from the municipio de Taxco, Guerrero, Mexico (also see Table A1).

### Isolation of *CYC2A* and *CYC2B*

We used the 77 degenerate primer pair combinations followed by exhaustive clone screening as described in Zhang et al. (2010, 2012) to isolate *CYC2*-like genes from our target species. These primers amplify *CYC*-like genes from many major Malpighiales clades, including especially the sister families of Malpighiaceae. *CYC2*-like amplicons spanning the TCP and R domains were obtained following our previous methods [described in Zhang et al. (2010)]. The PCR products were cloned, and more than 200 clones were screened for each sample to identify *CYC2* homologs.

### Sequence alignments and phylogenetic analyses

The newly acquired sequences of CYC2-like genes from *Microsteria sp., Rhynchophora phillipsonii, Philgamia glabrifolia, Sphedamnocarpus sp.(nov.), Lasiocarpus sp.*, and *Psychopterys dipholiphylla* were aligned with a previously available matrix including several ingroup accessions of Malpighiaceae, and the outgroup families, Elatinaceae, Centroplacaceae, and Oxalidaceae, by eye with reference to the translated amino acid sequences using MacClade 4.06 (Maddison and Maddison, 2003). The phylogeny in Figure 2 was based on amino acid sequence analyses. We applied the WAG + G model of amino acid evolution to the aligned CYC2 data set as determined by the AIC criterion in ProtTEST (Abascal et al., 2005). One thousand maximum likelihood bootstrap replicates were conducted using RAxML-VI-HPC (Stamatakis, 2006). Bayesian analyses were implemented in MrBayes ver. 3.1.2 (Huelsenbeck and Ronquist, 2001) under the mixed amino acid model. Analyses using nucleotide sequence data with third codon positions excluded under the best-fit model (“GTR + I + Γ”) for these data as determined using the Akaike Information Criterion (Akaike, 1973) in MODELTEST 3.06 (Posada and Crandall, 1998), yielded a topology nearly identical to the amino acid sequence data (not shown). One hundred ML bootstrap replicates were conducted with the optimal model of sequence evolution. Bayesian analyses were also conducted using the same model and default priors for the rate matrix, branch lengths, and gamma shape parameter. A Dirichlet distribution was used for the base frequency parameters and an uninformative prior was used for the starting tree topology. Four chains were initiated with a random starting tree and run for two million generations sampled every 1000 generations. Stationarity was determined using Tracer v1.4.1. (http://tree.bio.ed.ac.uk/software/tracer/). We sampled from the posterior distribution to calculate clade posterior probabilities following a burn-in of 1000 trees. DNA sequences of the newly acquired CYC2-like genes have been deposited in GenBank, under accession numbers KF514885-KF514893.

### Southern hybridization

Ten μg of genomic DNA was digested from *Microsteria sp*., *Rhynchophora phillipsonii, Philgamia glabrifolia*, and *Sphedamnocarpus sp. (nov.)* with restriction enzyme *Eco*RI, fractionated on 0.8% agarose gels, and blotted onto a positively charged nylon membrane (GE Healthcare Bio-Sciences Corp., Piscataway, NJ) following the protocol in Zhang et al. (2010). A fragment containing the 3′ end of the TCP domain and the variable region between the TCP and R domains was used as a template to synthesize probes for detecting *CYC2*-like genes. A mixture of *CYC2* sequences of *SphpCYC2A* and *SphpCYC2B* of *Sphedamnocarpus pruriens*, *SpheCYC2B* of *Sphedamnocarpus sp. (nov.)*, and *PgCYC2B* of *Philgamia glabrifolia* in equal molar concentration was used as a template to synthesize our ^32^P labeled probe to examine *Philgamia glabrifolia*, and *Sphedamnocarpus sp. (nov.);* and *CYC2* sequences of *TmCYC2A* and *TmCYC2B* of *Triaspis macropteron*, a member of the madagasikarioids and *MicroCYC2A* of *Microsteria sp*. to examine *Microsteria sp*., and *Rhynchophora phillipsonii*. We previously showed that the number of bands in the *Eco*RI digest is a reliable indicator of *CYC2* copy number (Zhang et al., 2010). Here, we identified a single band in the *Eco*RI digest for both *Microsteria sp*., and *Rhynchophora phillipsonii*, while two bands in the *Eco*RI digest for both *Philgamia glabrifolia*, and *Sphedamnocarpus sp. (nov.)* (Figure A2).

### RNA sample preparations

Floral buds from different developmental stages were prepared in liquid nitrogen in the field. They were grouped as small buds (~10–20% of full size buds); medium buds (~40–60% of full size buds); large buds (~70–90% of full size buds); and open flowers. All materials were preserved in cryogenic containers, and were processed in the lab using the RNAqueous kit (Ambion, Austin, TX, USA). Floral organs from medium to late stage samples, ~50–80% of flower bud size just before anthesis, were dissected in the lab from a single bud. These buds were dissected using the RNA*later*® -ICE Kit (Ambion-Applied Biosystems, Austin, TX, USA) The micro-dissected samples were processed using the RNAqueous Micro kit (Ambion, Austin, TX, USA). The details about this method described in Zhang et al. (2012).

### Reverse transcription (RT)-PCR

RT-PCR was performed using locus specific primers (Table A2) to examine the expression of *CYC2*. The sequence identity of RT-PCR fragments was further confirmed by sequencing.

### Quantitative RT-PCR

Samples from a single flower were analyzed in three separate qRT-PCRs. Three biological replicates, from three separate flowers, were conducted for each species. qRT-PCR reactions were conducted using PerfeCTa® SYBR® Green FastMix®, Low ROX™ (Quanta BioSciences, Inc., Gathersburg, MD) using the Stratagene Mx3005P QPCR System. Class I β*-tubulin* was used as a control to normalize the qRT-PCR (Oakley et al., 2007). *CYC2* expression levels were calculated relative to β*-tubulin* using the 2^−ΔΔCT^ method (Livak and Schmittgen, 2001). Details about the method and statistical analyses were described in Zhang et al. (2012).

### Conflict of interest statement

The authors declare that the research was conducted in the absence of any commercial or financial relationships that could be construed as a potential conflict of interest.

## Appendix

**Table A1:** **Species sampled in this study, with collection locations, voucher information, and *CYC2* loci**.

**Species**	**Lineage**	**Location**	**Voucher**	***CYC2* loci**	
				**2A**	**2B**
*Lasiocarpus sp*.	Radial ptilochaetoids	Municipio de Huitzuco de los Figueroa, Guerrero, Mexico	Steinmann and Davis 6147 (IEB)	*LasCYC2A*	*LasCYC2B*
*Microsteria sp*.	Madagasikarioids	Toliara, Madagascar	Zhang, Andrianatina, and Boufford 134 (A)	*MicroCYC2A*	–
*Philgamia glabrifolia* Arènes	Malagasy *Sphedamnocarpus*	Fianarantsoa, Mt. Ibity, Madagascar	Zhang, Andrianatina, and Boufford 129 (A)	–	*PgCYC2B*
*Psychopterys dipholiphylla* (Small) W. R. Anderson and S. Corso	*Psychopterys dipholiphylla*	Municipio de Eduardo Neri, Guerrero, Mexico	Steinmann and Davis 6143 (IEB)	*PdCYC2A-1, -2*	*PdCYC2B*
*Rhynchophora phillipsonii* W. R. Anderson	Madagasikarioids	Toliara, Madagascar	Zhang, Andrianatina, and Boufford 144 (A)	*RpCYC2A*	–
*Sphedamnocarpus sp.(nov.)*	Malagasy *Sphedamnocarpus*	Fianarantsoa, Isalo National Park, Madagascar	Zhang, Andrianatina, and Boufford 132 (A)	–	*SpheCYC2B*

*Note: A, Arnold Herbarium, Harvard University Herbaria; IEB, Instituto de Ecología, A.C.; –, gene copy can not be detected by degenerate PCR on genomic DNA or cDNA*.>

**Table A2:** **qRT-PCR primer sequences, amplicon size, and amplification efficiency**.

**Name**	**Taxa**	**Forward (5^′^ to 3^′^)**	**Reverse (5^′^ to 3^′^)**	**Amplicon size (bp)**	**Amplification efficiency (%)**
*LasCYC2A*	*Lasiocarpus sp*.	TTAGGGTTTGACAGGGCAAGT	TGCTTAGCAACAAGTGGAATTT	251	101
*LasCYC2B*	*Lasiocarpus sp*.	AGAGCAAGCAAAACCCTTGA	GAAGCTTCTTGCAATTTTTGAGA	233	84
*LasTUB1*	*Lasiocarpus sp*.	TCAGGGAGGAGTACCCTGATAGA^*^	GCAAGTGACACCACTCATTGTC^*^	251	81
*MicroCYC2A*	*Microsteria sp*.	AAACCCTTGAATGGCTCCTT	GCTTAGCAACAAGTGGGGTTT	228	81
*MicroTUB1*	*Microsteria sp*.	TCAGGGAGGAGTACCCTGATAGA^*^	GCAAGTGACACCACTCATTGTC^*^	251	105
*PdCYC2A*	*Psychopterys dipholiphylla*	CTCGTCATGTGACCAAAAAGA	TGCTTAGCAACAAGTGGGATT	104	88
*PdCYC2B*	*Psychopterys dipholiphylla*	AAAACCCTTGAATGGCTTCTTAC	GATCGCCCGTCAAGTTACTC	127	89
*PdTUB1short*	*Psychopterys dipholiphylla*	TGTGTTTCCATCACCAAAGG	GCAGATATCGTACAAAGCCTCA	130	110
*SpheCYC2B*	*Sphedamnocarpus sp.(nov.)*	AAAGCAAGCAAAACCCTTGA	CCGAAGCTTTTTGCAATTTT	238	–

* *These primers were published in Zhang et al. (2012); bp, base pairs*.
